# Predictors of developing severe COVID-19 among hospitalized patients: a retrospective study

**DOI:** 10.3389/fmed.2024.1494302

**Published:** 2025-01-14

**Authors:** Hussain Abduljaleel Alkhalifa, Ehab Darwish, Zaenb Alsalman, Aman Alfaraj, Abdullah Alkhars, Fatimah Alkhalifa, Mohammed Algaraash, Ahmed Mohammed Elshebiny, Emad Alkhoufi, Khaled Mohamed Amin Elzorkany

**Affiliations:** ^1^Internal Medicine Department, College of Medicine, King Faisal University, Al-Ahsa, Saudi Arabia; ^2^Family and Community Medicine Department, College of Medicine, King Faisal University, Al-Ahsa, Saudi Arabia; ^3^Internal Medicine Department, King Fahad Specialist Hospital, Dammam, Saudi Arabia; ^4^Department of Pediatric, King Fahad Medical City, Riyadh, Saudi Arabia; ^5^Pathology Department, King Fahad Specialist Hospital, Dammam, Saudi Arabia; ^6^Internal Medicine Department, Prince Saud Bin Jalawi Hospital, Al-Ahsa, Saudi Arabia

**Keywords:** COVID-19, SARS-CoV-2, severe, risk factors, outcome, epidemiology

## Abstract

**Background:**

COVID-19 poses a significant threat to global public health. As the severity of SARS-CoV-2 infection varies among individuals, elucidating risk factors for severe COVID-19 is important for predicting and preventing illness progression, as well as lowering case fatality rates. This work aimed to explore risk factors for developing severe COVID-19 to enhance the quality of care provided to patients and to prevent complications.

**Methods:**

A retrospective study was conducted in Saudi Arabia’s eastern province, including all COVID-19 patients aged 18 years or older who were hospitalized at Prince Saud Bin Jalawi Hospital in July 2020. Comparative tests as well as both univariate and multivariate logistic regression analyses were performed to identify risk factors for developing severe COVID-19 and poor outcomes.

**Results:**

Based on the comparative statistical tests patients with severe COVID-19 were statistically significantly associated with older age and had higher respiratory rate, longer hospital stay, and higher prevalence of diabetes than non-severe cases. They also exhibited statistically significant association with high levels of potassium, urea, creatinine, lactate dehydrogenase (LDH), D-dimer, and aspartate aminotransferase (AST). The univariate analysis shows that having diabetes, having high severe acute respiratory infection chest X-ray scores, old age, prolong hospitalization, high potassium and lactate dehydrogenase, as well as using insulin, heparin, corticosteroids, favipiravir or azithromycin were all statistically significant associated with severe COVID-19. However, after adjustments in the multivariate analysis, the sole predictor was serum LDH (*p* = 0.002; OR 1.005; 95% CI 1.002–1.009). In addition, severe COVID-19 patients had higher odds of being prescribed azithromycin than non-severe patients (*p* = 0.001; OR 13.725; 95% CI 3.620–52.043). Regarding the outcomes, the median hospital stay duration was statistically significantly associated with death, intensive care unit admission (ICU), and mechanical ventilation. On the other hand, using insulin, azithromycin, beta-agonists, corticosteroids, or favipiravir were statistically significantly associated with reduced mortality, ICU admission, and need of mechanical ventilation.

**Conclusion:**

This study sheds light on numerous parameters that may be utilized to construct a prediction model for evaluating the risk of severe COVID-19. However, no protective factors were included in this prediction model.

## Introduction

In December 2019, the first case of COVID-19 was reported in Wuhan, a Chinese city in the province of Hubei (1). Its causative agent, SARS-CoV-2, is among the deadliest coronaviruses, alongside SARS-CoV-1, which causes severe acute respiratory syndrome (SARS), and MERS-CoV, which causes Middle East respiratory syndrome (MERS) (2, 3). However, the morbidity and mortality of COVID-19 are far greater than those of SARS and MERS (3).

COVID-19 has a wide spectrum of clinical manifestations, from asymptomatic to critical and life-threatening (4). Severe cases usually require artificial ventilation and intensive care unit (ICU) admission. Additionally, the disease’s case fatality rate is estimated to range from 3.4 to 11% (5, 6). Because certain populations have a higher chance of developing adverse outcomes, uncovering the risk factors that promote severe disease is important for predicting and preventing illness progression, as well as lowering the case fatality rate (6). Previous studies have constructed predictive models using various risk factors to identify high-risk groups that may develop severe COVID-19 infection. These factors range from demographic factors such as age, sex, and ethnicity to comorbidities and laboratory results (7). However, only a few published papers have investigated risk factors for severe SARS-CoV-2 specifically among the Saudi population in the Eastern province (8, 9). According to Al Dossary’s study, age, gender, and ethnicity are key predictors of COVID-19 severity; however, this study did not look at the relationship between laboratory parameters and COVID-19 severity or prognosis (8). Moreover, Ansari et al., who studied 1,444 hospitalized COVID-19 patients, found that old age, the presence of underlying cardiovascular conditions, an abnormal white blood count, and abnormal blood urea nitrogen levels are independent predictors of mortality, but his study did not account for disease severity or other outcomes such as ICU admission and mechanical ventilation requirement (9). Despite having larger sample sizes and statistically significant results, these studies had some limitations and still there is no conclusive data available.

Geographic variation may potentially contribute to differences in COVID-19 risks and severity (10). The reasons behind these differences are unknown, although they have been linked to a complex and interconnected patterning of several elements (10). Furthermore, in Saudi Arabia, the existence of religious sites that attract millions of people each year raises the risk of possible outbreaks (11). Population-based data could aid the identification of risk factors, which could then be used to optimize COVID-19 case management, provide more individualized guidance to patient groups, and reduce case fatalities (12, 13). Therefore, this work aimed to explore risk factors for developing severe COVID-19 as well as look at determinants of mortality, ICU admission and mechanical ventilation requirement to enhance the quality of care provided to patients and to prevent complications.

## Materials and methods

### Study design and participants

A retrospective cohort study was conducted in Saudi Arabia’s eastern province, including all COVID-19 patients aged 18 years or older who were hospitalized at Prince Saud Bin Jalawi Hospital in July 2020. Diagnoses were confirmed by polymerase chain reaction (PCR) testing. Patients who did not have an electronic medical record were excluded.

Data collected from the medical record system included sociodemographic data of the patient, basic clinical and laboratory data, medication history, and hospital course (length of hospital stay, severe COVID-19, and clinical outcome), with all patients being tracked until discharge or death.

### Definitions

Chest X-ray clinical findings were graded based on the severe acute respiratory infection (SARI) chest X-ray severity scoring system as 1: normal; 2: patchy atelectasis and/or hyperinflation and/or bronchial wall thickening; 3: focal consolidation; 4: multifocal consolidation; and 5: diffuse alveolar changes (14). Then patients were divided into two groups based on their SARI score; low score (1-3) and high score (4-5).

Patients were categorized according to disease severity into severe and non-severe cases. Severe COVID-19 was defined as per the Saudi Ministry of Health Protocol for COVID-19, particularly taking an oxygen saturation below 93% in room air or a respiratory rate above 30/min as the determining criteria (15).

Clinical outcome was assessed in terms of percent mortality, mechanical ventilation, and ICU admission.

### Statistical analysis

Data were analyzed using the Statistical Package for Social Sciences (SPSS) for Windows, version 22 (SPSS Inc. Chicago, IL, USA). Shapiro–Wilk and Kolmogorov–Smirnov tests were used to assess the normality of continuous data. Continuous variables were expressed as mean and standard deviation and categorical data as numbers and percentages. The comparative tests (Chi-square (χ2) and Fisher’s exact tests for categorical variables, and independent t-test and Mann–Whitney U test for quantitative variables) were used to identify significant factors for COVID-19 disease severity as well as clinical outcome (mortality, mechanical ventilation, and ICU admission). The following independent variables were interpreted as continuous data; age, length of hospitalization, hemoglobin, leukocyte, platelets, potassium, sodium, AST, albumin, urea, creatinine, lactate dehydrogenase, creatine Kinase, and d-dimer, while other data interpreted as categorical data; Sex, ICU admission, Mechanical ventilation, DM, HTN, Respiratory diseases, Cardiovascular disease, Chronic kidney disease, SARI CXR Severity, Insulin, Heparin, Corticosteroids, Favipiravir, and Azithromycin. These variables were chosen based on the literature review and their clinical relevance. Then both univariate and multivariate logistic regression analyses were performed to identify COVID-19 disease severity predictors. Variables with clinical relevance and statistical significance from the comparative tests were included in a univariate analysis. However, even if a variable is statistically significant in comparative analysis, it may be excluded from further analysis based on clinical judgment, data quality issues, or if it is deemed redundant or highly collinear with other variables. Furthermore, variables with statistically significant association on univariate analysis were included in a multivariable regression model. This step-by-step strategy aims to refine relevant predictors for future multivariate modelling, hence improving the robustness of our findings. All statistical analyses were based on two-sided hypothesis tests with a significance level of *p* < 0.05.

### Ethical consideration

The Institutional Review Board at The King Fahad Hospital approved this study (IRB KFHH No. H-05-HS-065) and waived patient consent due to the study’s retrospective nature. The study was performed according to the Helsinki Declaration, and all data were collected, coded, and analyzed to ensure data integrity and patient privacy.

## Results

### Epidemiological and clinical characteristics

A total of 251 COVID-19 patients were included in this study. The majority (57%) were male, and 39.8% were over the age of 60 years. Hypertension and diabetes mellitus constituted the most common comorbidities (51.4 and 49.8% respectively). Hospital admission periods ranged 1–31 days with a median of 7 days; in addition, 15.9% of patients were admitted to the ICU, 19.9% suffered mortality, 18.7% required mechanical ventilation, and around two-thirds (70.1%) had severe COVID-19. Heparin (88.8%), azithromycin (85.3%), cephalosporin (84.5%), corticosteroids (71.7%), and favipiravir (47.4%) were the most common prescribed drugs (Table 1).

**Table 1:** Characteristics of patients (No. = 251).

Parameter		No. (%)
Age (Years)	18–40	49 (19.5)
	40–60	102 (40.6)
	>60	100 (39.8)
Gender	Male	143 (57.0)
	Female	108 (43.0)
Vital Signs	Pulse Rate > 100 bpm	111 (44.2)
	Systolic BP < 90	6 (2.4)
	Diastolic BP <60	38 (15.1)
	Temperature > 38.0°C	61 (24.3)
	Respiratory Rate > 20 bpm	92 (36.7)
	Oxygen Saturation < 95%	214 (85.3)
Hospital Course	Days of Stay (Median, Range)	7, 1–31
	ICU Admission	40 (15.9)
	Intubation and Mechanical Ventilation	47 (18.7)
	Severe COVID-19	176 (70.1)
	Mortality	50 (19.9)
Comorbidities	Diabetes Mellitus	125 (49.8)
	Hypertension	129 (51.4)
	Cardiovascular Disease	32 (12.7)
	Chronic Kidney Disease	12 (4.8)
	Asthma/COPD	15 (6.0)
	Sickle Cell Disease	7 (2.8)
	Malignancy	5 (2.0)
	Liver Disease	2 (0.8)
	Stroke	10 (4)
Medications	ACEi/ARBs	69 (27.5)
	Potassium-sparing Diuretics	9 (3.6)
	Insulin	121 (48.2)
	Beta Agonist	50 (19.9)
	Beta Blocker	53 (21.1)
	Heparin	223 (88.8)
	Antiplatelet	59 (23.5)
	Corticosteroids	180 (71.7)
	Hydroxychloroquine	41 (16.3)
	Ribavirin	34 (13.5)
	Lopinavir/Ritonavir	55 (21.9)
	Interferon B	54 (21.5)
	Favipiravir	119 (47.4)
	Interleukin-6 Inhibitors	21 (8.4)
	Vasopressors	40 (15.9)
	Linezolid	12 (4.8)
	Tetracycline	16 (6.4)
	Azithromycin	214 (85.3)
	Cephalosporin	212 (84.5)
	Tazocin	53 (21.1)
	Carbapenems	44 (17.5)
	Fluoroquinolones	22 (8.8)
	Vancomycin	11 (4.4)
SARI CXR Score (No.=218)	1-3	53 (24.3)
	4-5	165 (75.7)
Laboratory findings (Median, Range)	Hemoglobin (g/dL)	12.5, 4.9–17.9
	Hematocrit	38.7, 5.01–56.3
	Leukocyte (1,000 cells/μL)	6.07, 2.06–28.49
	Platelets (1,000 platelets/μL)	235, 20–668
	Potassium (mmol/L)	4.45, 2.82–8.25
	Sodium (mmol/L)	138.5, 124–177
	Creatinine (µmol/L)	85, 18–3,805
	Urea (mmol/L)	5.8, 1.5–85
	Albumin (g/L)	30.04, 16.7–44.7
	Lactate Dehydrogenase (U/L)	339, 109–847
	creatine Kinase (µmol/L)	115, 20–5,916
	D-dimer (mg/L)	1.05, 0.1–21.9
	Ferritin (ng/mL)	745, 20–2000
	AST (U/L)	42, 6–835

Abbreviations: ICU, Intensive care units; COPD, Chronic obstructive pulmonary disease; ACEi, angiotensin converting enzyme; ARBs, Angiotensin receptor blockers; AST, aspartate aminotransferase.

Regarding radiology, more than 75% of patients had score 4–5 on the SARI CXR system. In laboratory results, the median levels of hemoglobin, white blood cells, and platelets were 12.5 gm/dL, 6.07 × 10^9^/L, and 235 × 10^9^/L respectively, while median levels of potassium, urea, and creatinine were 4.45 mmol/L, 5.8 mmol/L, and 85 μmol/L, respectively, (Table 1).

### Factors associated with the severity of COVID-19

Based on comparative statistical tests, severe COVID-19 was statistically significantly associated with older age, high respiratory rate, prolonged hospital stay, and diabetes mellitus; the use of insulin, heparin, corticosteroids, favipiravir, or azithromycin; and higher levels of potassium, urea, creatinine, lactate dehydrogenase (LDH), D-dimer, and aspartate aminotransferase (AST), as well as high SARI CXR scores (Tables 2, 3). Of note, respiratory rate was eliminated from the univariate analysis, although being statistically significant in the comparative analysis, because it is used to assess illness severity and is related to mechanical ventilation.

**Table 2:** Clinical data according to COVID-19 severity (No. = 251).

Variable		COVID-19 Severity No. (%)		*p*-value
		Non-severe (No. = 75)	Severe (No. = 176)	
Age (Years)	18–40	22 (44.9)	27 (55.1)	
	40–60	33 (32.4)	69 (67.6)	0.006*
	>60	20 (20)	80 (80)	
Gender	Male	38 (26.6)	105 (73.4)	0.181
	Female	37 (34.3)	71 (65.7)	
Vital Signs	Pulse Rate > 100 bpm	33 (29.7)	78 (70.3)	0.538
	Systolic BP < 90	3 (50)	30 (50)	0.081
	Diastolic BP <60	8 (21.1)	30 (78.9)	0.329
	Temperature > 38.0°C	17 (27.9)	44 (72.1)	0.844
	Respiratory Rate > 20 bpm	18 (19.6)	74 (80.4)	0.004*
Hospital Course	Days of Stay (mean ± SD)	7.26 ± 5.876	9.61 ± 5.705	0.004*
	ICU Admission (yes)	8 (20)	32 (80)	0.094
	Mechanical Ventilation (yes)	10 (21.3)	37 (78.7)	0.103
	Mortality (yes)	11 (22)	39 (78)	0.116
Comorbidities (YES)	Diabetes Mellitus	26 (20.8)	99 (79.2)	0.001*
	Hypertension	34 (26.4)	95 (73.6)	0.132
	Cardiovascular disease	11 (34.4)	21 (65.6)	0.343
	Chronic Kidney Disease	2 (16.7)	10 (83.3)	0.249
	Asthma/COPD	4 (26.7)	11 (73.3)	0.518
	Sickle Cell Disease	3 (42.9)	4 (57.1)	0.349
	Malignancy	3 (60)	2 (40)	0.156
	Liver Disease	1 (50)	1 (50)	0.505
	Stroke	3 (30)	7 (70)	0.618
Medications (YES)	ACEi/ARBs	18 (26.1)	51 (73.9)	0.535
	Potassium-sparing Diuretics	4 (44.4)	5 (55.6)	0.323
	Insulin	22 (18.2)	99 (81.8)	<0.001*
	Beta Agonist	10 (20.4)	39 (79.6)	0.12
	Beta Blocker	17 (32.1)	36 (67.9)	0.666
	Heparin	58 (26.1)	164 (73.9)	<0.001*
	Antiplatelet	17 (28.8)	42 (71.2)	0.872
	Corticosteroids	43 (24)	136 (76)	0.001*
	Hydroxychloroquine	13 (31.7)	28 (68.3)	0.789
	Ribavirin	12 (36.4)	21 (63.6)	0.388
	Lopinavir/Ritonavir	16 (29.6)	38 (70.4)	0.952
	Interferon B	14 (25.9)	40 (74.1)	0.506
	Favipiravir	24 (20.3)	94 (79.7)	0.002*
	Interleukin-6 Inhibitors	5 (23.8)	16 (76.2)	0.624
	Vasopressor	10 (25)	30 (75)	0.572
	Linezolid	3 (25)	9 (75)	0.701
	Tetracycline	4 (25)	12 (75)	0.753
	Azithromycin	55 (25.7)	159 (74.3)	<0.001*
	Cephalosporin	59 (28.0)	152 (72)	0.097
	Tazocin	13 (24.5)	40 (75.5)	0.33
	Carbapenems	12 (27.9)	31 (72.1)	0.746
	Fluoroquinolones	6 (28.6)	15 (71.4)	0.885
	Vancomycin	4 (36.4)	7 (63.6)	0.635

*Significant at *p*-value ≤ 0.05.

**Table 3:** Laboratory data according to COVID-19 severity (No. = 251).

Variable	COVID-19 Severity		*P*-value
	Non-severe (No. = 75)	Severe (No. = 176)	
Hemoglobin (g/dL)	12.71 ± 2.34	12.38 ± 2.37	0.295
Hematocrit	38.7 ± 6.89	37.94 ± 7.42	0.457
Leukocyte (1,000 cells/μL)	7.44 ± 4.69	7.36 ± 4.46	0.887
Platelets (1,000 platelets/μL)	244.01 ± 114.48	256.57 ± 108.29	0.469
Potassium (mmol/L)	4.37 ± 0.75	4.59 ± 0.73	0.006*
Sodium (mmol/L)	139.1 ± 6.14	138.2 ± 6.18	0.309
Creatinine (µmol/L)	90.3 ± 56.57	112.04 ± 92.39	0.035*
Urea (mmol/L)	7.1 ± 7.17	9.12 ± 10.18	0.007*
Albumin (g/L)	30.29 ± 6.43	29.91 ± 5.88	0.624
Lactate Dehydrogenase (U/L)	299.8 ± 129.69	391.19 ± 158.25	<0.001*
creatine Kinase (μmol/L)	241.29 ± 396.94	358.35 ± 704.80	0.052
D-dimer (mg/L)	0.79 ± 0.43	2.98 ± 4.29	0.002*
Ferritin (ng/mL)	925.12 ± 687.92	1021.46 ± 721.55	0.727
AST (U/L)	52.86 ± 32.01	66.20 ± 119.8	0.039*
SARI CXR score (4-5)	39 (23.6)	126 (76.4)	<0.001*

^*^Significant at *p*-value ≤ 0.05.

The univariate analysis shows that patients with diabetes mellitus had 2.423 times higher odds of developing severe COVID-19 compared to those without diabetes. Similarly, those with high SARI CXR scores had 3.618 times higher odds of having severe COVID-19. Moreover, old age, prolong hospitalization, high potassium and lactate dehydrogenase, as well as using insulin, heparin, corticosteroids, favipiravir or azithromycin were all significantly associated with severe COVID-19 (Table 4). However, after adjustments in the multivariate analysis, the sole predictor was serum LDH (*p* = 0.002; OR 1.005; 95% CI 1.002–1.009). Additionally, azithromycin was statistically significantly more prescribed in severe COVID-19 cases (*p* = 0.001; OR 13.725; 95% CI 3.620–52.043) (Table 4).

**Table 4:** Predictors of severe COVID-19 disease.

Variable	Univariable		Multivariable	
	Logistic analysis		Logistic analysis	
	OR (95% CI)	*P*-value	OR (95% CI)	*P*-value
Age	1.028 (1.010–1.046)	0.002*	1.014 (0.989–1.040)	0.263
Sex (Male)	0.694 (0.403–1.196)	0.189		
Length of hospitalization	1.078 (1.019–1.140)	0.008*	1.059 (0.981–1.143)	0.141
ICU admission (Yes)	1.861 (3.24–10.5)	0.141		
Mechanical ventilation (Yes)	1.730 (0.811–3.693)	0.156		
DM (Yes)	2.423 (1.383–4.427)	0.002*	1.995 (0.670–5.941)	0.215
HTN (Yes)	1.414 (0.822–2.434)	0.211		
Asthma/COPD (Yes)	1.183 (0.364–3.842)	0.779		
Cardiovascular disease (Yes)	0.788 (0.359–1.279)	0.553		
Chronic kidney disease (Yes)	2.199 (0.470–10.287)	0.317		
SARI CXR score (4–5)	3.618 (1.893–6.917)	<0.001*	1.821 (0.753–4.404)	0.184
Hemoglobin	0.943 (0.837–1.064)	0.34		
Leukocyte	0.991 (0.932–1.054)	0.781		
Platelets	1.001 (0.998–1.003)	0.585		
Potassium	1.524 (1.016–2.287)	0.042*	0.786 (0.440–1.404)	0.416
Sodium	0.978 (0.935–1.023)	0.33		
AST	0.998 (0.994–1.002)	0.325		
Albumin	1.003 (0.926–1.086)	0.941		
Urea	1.033 (0.988–1.079)	0.149		
Creatinine	1.001 (0.999–1.003)	0.463		
Lactate Dehydrogenase	1.005 (1.002–1.007)	<0.001*	1.005 (1.002–1.009)	0.002*
creatine Kinase	1 (1–1.001)	0.305		
D-dimer	4.766 (0.998–22.769)	0.051		
Insulin (Yes)	3.120 (1.744–5.583)	<0.001*	1.571 (0.534–4.620)	0.412
Heparin (Yes)	4.492 (2.072–11.785)	<0.001*	2.317 (0.646–8.312)	0.197
Corticosteroids (Yes)	2.590 (1.441–4.653)	<0.001*	1.190 (0.475–2.980)	0.711
Favipiravir (Yes)	2.350 (1.335–4.13)	0.003*	1.248 (0.527–2.954)	0.614
Azithromycin (Yes)	3.614 (1.750–7.464)	<0.001*	13.725 (3.620–52.043)	<0.001*

^*^Significant at *p*-value ≤ 0.05.

The comparative statistical analysis for demographic and clinical factors with COVID-19 outcomes (mortality, mechanical ventilation, and ICU admission) is presented in Table 5. The median hospital stay duration was statistically significantly associated with all three outcomes. Patients who died had a median stay of 11 days, compared to 7 days for those who survived (*p* = 0.001). Similarly, patients requiring intubation had a median stay of 15 days, compared to 6 days for those who did not require intubation (*p* < 0.001). Those admitted to the ICU had a median stay of 17.5 days, while those not admitted to the ICU had a median stay of 6 days (*p* < 0.001). Regarding patients’ comorbidities, chronic kidney disease was statistically significantly associated with increase mortality (*p* = 0.001).

**Table 5:** Association of clinical factors with the COVID outcomes (No. = 251).

Variable		Mortality		*P-*value	Intubation		*P-*value	ICU		*P-*value
		Yes	No		Yes	No		Yes	No	
		No. (%)	No. (%)		No. (%)	No. (%)		No. (%)	No. (%)	
AGE (years)	18–40	9 (18.4)	40 (81.6)	0.408	8 (16.3)	41 (83.7)	0.886	7 (14.3)	42 (85.7)	0.628
	40–60	17 (16.7)	85 (83.3)		20 (19.6)	82 (80.4)		19 (18.6)	83 (81.4)	
	>60	24 (24)	76 (76)		19 (19)	81 (81)		14 (14)	86 (86)	
Gender	Female	19 (17.6)	89 (82.4)	0.422	17 (15.7)	91 (84.3)	0.292	3 (12)	95 (88)	0.142
	Male	31 (21.7)	112 (78.3)		30 (21)	13 (79)		27 (18.9)	116 (81.1)	
Vital Signs	Heart Rate > 100 bpm	25 (22.5)	86 (77.5)	0.358	27 (24.3)	84 (75.7)	0.043*	26 (23.4)	85 (76.6)	0.004*
	Systolic BP < 90	3 (50)	3 (50)	0.062	2 (33.3)	4 (66.7)	0.353	1 (16.7)	5 (83.3)	0.961
	Diastolic BP <60	12 (31.6)	26 (68.4)	0.051	9 (23.7)	29 (76.3)	0.395	5 (13.2)	33 (86.8)	0.611
	Temperature > 38.0°	10 (16.4)	51 (83.6)	0.428	11 (18)	50 (82)	0.873	12 (19.7)	49 (80.3)	0.36
	Respiratory Rate > 20 bpm	28 (30.4)	64 (69.6)	0.002*	27 (29.3)	65 (70.7)	0.001*	22 (23.9)	70 (76.1)	0.009*
	Oxygen Saturation < 93%	38 (22)	135 (78)	0.185	37 (21.4)	136 (78.6)	0.137	31 (17.9)	142 (82.1)	0.085
	Days of Stay (median, IQR)	11 (12)	7 (5)	0.001*	15 (12)	6 (4)	<0.001*	17.5 (8)	6 (4)	<0.001*
	SARI CXR score (4–5)	40 (24.2)	125 (75.8)	0.02*	37 (22.4)	128 (77.6)	0.016*	33 (18.8)	134 (81.2)	0.022*
	Severe COVID-19	38 (21.8)	136 (78.2)	0.207	37 (21.3)	137 (78.7)	0.154	31 (17.8)	143 (82.2)	0.095
Comorbidities	Diabetes Mellitus	30 (24)	95 (76)	0.107	28 (22.4)	97 (77.6)	0.137	21 (16.8)	104 (83.2)	0.71
	Hypertension	30 (23.3)	99 (76.7)	0.174	26 (20.2)	103 (79.8)	0.55	19 (14.7)	110 (85.3)	0.591
	Cardiovascular disease	8 (25)	24 (75)	0.441	6 (18.8)	26 (81.2)	0.997	4 (12.5)	28 (87.5)	0.57
	Chronic Kidney Disease	7 (58.3)	5 (41.7)	0.001*	5 (41.7)	7 (58.3)	0.037*	3 (25)	9 (75)	0.379
	Asthma/COPD	2 (13.3)	13 (86.7)	0.51	2 (13.3)	13 (86.7)	0.581	0 (0)	15 (100)	0.082
	Sickle Cell Disease	0 (0)	7 (100)	0.181	0 (0)	7 (100)	0.198	0 (0)	7 (100)	0.243
	Malignancy	2 (40)	3 (60)	0.259	2 (40)	3 (60)	0.22	2 (40)	3 (60)	0.139
	Liver disease	0 (0)	2 (100)	0.483	0 (0)	2 (100)	0.5	0 (0)	2 (100)	0.535
Medications	ACEi/ARBs	14 (20.3)	55 (79.7)	0.912	10 (14.5)	59 (85.5)	0.299	6 (8.7)	63 (91.3)	0.07
	K-sparing diuretics	6 (66.7)	3 (33.3)	<0.001*	6 (66.7)	3 (33.3)	<0.001*	3 (33.3)	6 (66.7)	0.128
	Insulin	36 (29.8)	85 (70.2)	<0.001*	37 (30.6)	84 (69.4)	<0.001*	28 (23.1)	93 (76.9)	0.002*
	Beta agonist	19 (38)	31 (62)	<0.001*	21 (42)	29 (58)	<0.001*	16 (32)	34 (68)	<0.001*
	Beta blocker	22 (41.5)	31 (58.5)	<0.001*	16 (30.2)	37 (69.8)	0.015*	12 (22.6)	41 (77.4)	0.098
	Heparin	45 (20.2)	178 (79.8)	0.619	42 (18.8)	181 (81.2)	0.73	36 (16.1)	187 (83.9)	0.284
	Antiplatelet	14 (23.7)	45 (76.3)	0.38	12 (20.3)	47 (79.7)	0.685	8 (13.6)	51 (86.4)	0.667
	Corticosteroids	43 (23.9)	137 (76.1)	0.008*	42 (23.3)	138 (76.7)	0.002*	36 (20)	144 (80)	0.001*
	Hydroxychloroquine	5 (12.2)	36 (87.8)	0.183	5 (12.2)	36 (87.8)	0.252	6 (14.6)	35 (85.4)	0.893
	Ribavirin	11 (32.4)	23 (67.6)	0.047*	9 (26.5)	25 (73.5)	0.201	8 (23.5)	26 (76.5)	0.153
	Lopinavir _rotinvir	14 (25.5)	41 (74.5)	0.229	11 (20)	44 (80)	0.754	11 (20)	44 (80)	0.275
	Interferon-B	15 (27.8)	39 (72.2)	0.094	12 (22.2)	42 (77.8)	0.432	10 (18.5)	44 (81.5)	0.461
	Favipiravir	32 (26.9)	87 (73.1)	0.006*	35 (29.4)	84 (70.6)	<0.001*	31 (26.1)	88 (73.9)	<0.001*
	Interleukin 6 inhibitors	17 (81)	4 (19)	<0.001*	17 (81)	4 (19)	<0.001*	16 (76.2)	5 (23.8)	<0.001*
	Vasopressors	38 (95)	2 (5)	<0.001*	35 (87.5)	5 (12.5)	<0.001*	24 (60)	16 (40)	<0.001*
	Linezolid	11 (91.7)	1 (8.3)	<0.001*	11 (91.7)	1 (8.3)	<0.001*	7 (58.3)	5 (41.7)	<0.001*
	Tetracycline	2 (12.5)	14 (87.5)	0.438	2 (12.5)	14 (87.5)	0.529	4 (25)	12 (75)	0.284
	Azithromycin	37 (17.3)	177 (82.7)	0.009*	35 (16.4)	179 (83.6)	0.042*	28 (13.1)	186 (86.9)	0.008*
	Cephalosporin	38 (17.9)	174 (82.1)	0.053	38 (17.9)	174 (82.1)	0.647	33 (15.6)	179 (84.4)	0.972
	Tazocin	27 (50.9)	26 (49.1)	<0.001*	25 (47.2)	28 (52.8)	<0.001*	19 (35.8)	34 (64.2)	<0.001*
	Carbapenems	26 (59.1)	18 (40.9)	<0.001*	28 (63.6)	16 (36.4)	<0.001*	25 (56.8)	19 (43.2)	<0.001*
	Fluoroquinolons	9 (40.9)	13 (59.1)	0.010*	8 (36.4)	14 (63.6)	0.023*	6 (27.3)	16 (72.7)	0.114
	Vancomycin	5 (45.5)	6 (54.5)	0.031*	7 (63.6)	4 (36.4)	<0.001*	5 (45.5)	6 (54.5)	0.005*

^*^Significant at *p*-value ≤ 0.05.

Patients treated with Interleukin 6 inhibitors, Vasopressors, Carbapenems, or Linezolid had a statistically significant association with increased poor outcomes, including death, intubation, and ICU admission (*p* < 0.001). Moreover, using K-sparing diuretics was statistically significantly associated with increased mortality and need of intubation (*p* < 0.001). On the other hand, using insulin, azithromycin, beta-agonists, corticosteroids, or favipiravir was statistically significantly associated with reduced all these poor outcomes. Tazocin was statistically significantly associated with increased mortality but reduced intubation and ICU admission (*p* < 0.001). Furthermore, patients on Vancomycin were statistically significantly associated with increased intubation, although they had decreased mortality and ICU admission. Using beta blockers was statistically significantly associated with decreased mortality and the requirement for intubation.

## Discussion

The COVID-19 pandemic has challenged healthcare systems in both developed and developing countries, with a wide spectrum of clinical symptoms and consequences (16). Currently based on the WHO, COVID-19 is an established health concern that requires long-term care, rather than a worldwide health emergency (17). This study shed light on different demographic and clinical characteristics associated with severe COVID-19 which might assist in minimizing adverse consequences. Findings from this study found that old age, high respiratory rate, long hospital stay, and diabetes as well as high levels of potassium, urea, creatinine, LDH, D-dimer, and AST all were statistically significantly associated with severe COVID-19. However, when these factors were merged for further analysis, the sole predictor was serum LDH, possibly due to multicollinearity and confounding. In addition, azithromycin was more prescribed in severe cases, which is likely associated with severe COVID-19. Regarding the clinical outcomes, the median hospital stay duration was statistically significantly associated with increased mortality, ICU admission, and mechanical ventilation requirements. However, using azithromycin, corticosteroids, or favipiravir was statistically significantly associated with reduced mortality, ICU admission, and need for mechanical ventilation.

In the current study, the median period of hospital stay was seven days, which aligns with a recent Saudi study (18). Additionally, approximately two-thirds (70.1%) of our patients had severe disease, 15.9% were admitted to the ICU, and the mortality was 19.9%. In a previous Chinese study, 81% of cases were mild, 14% were severe and required ventilation in the ICU, 5% were critical and involved respiratory failure, septic shock, and/or multiple organ dysfunction and the overall case-fatality rate was 2.3% (19). The greater percentage of severe cases in the present study may be attributed to it exclusively including hospitalized patients. Another study in New York assessed the outcome of 2,634 patients who were discharged or had died at their study end point. Of the 2,634 patients; 14.2% were treated in the ICU, 12.2% received invasive mechanical ventilation, and 21% died (20). Likewise, in a 2020 study of hospitalized patients in Germany, 22% died (21).

Numerous factors contribute to poor outcomes in patients with COVID-19-infection. This study found COVID-19 severity to be higher in older patients, consistent with the findings of Wang et al. and Zhang et al., who reported increased age to be one of the most commonly stated demographic factors that increases the probability of a severe course of disease and poor outcomes (22, 23). These findings could be attributed to the greater likelihood of comorbidities and weakening of the immune system as people age (24).

Regarding gender, most COVID-19 patients in this study were male (57%), which may confirm earlier reports that male sex is a risk factor for a positive SARS-CoV-2 test (25–27). This difference in viral attachment between men and women may be due to biological variances, differences in immunological responses, sex hormones, or a confluence of risky behaviors or lifestyle choices (28). Notably, Assiri et al. observed the conflicting finding that women were more likely to have a positive SARS-CoV-2 test than men. This discrepancy is expected because of the greater recruitment of female patients from the affiliated women’s university (Princess Nourah bint Abdulrahman University) in that study (18). On the other hand, our study, found no sex disparity in the severity and outcome of COVID-19 patients which aligns with Albishi et al. study in Jeddah, Saudi Arabia (29).

The most prevalent comorbidities among COVID-19 participants in our study were hypertension (51.4%) and diabetes (49.8%). These findings agree with earlier reports in Saudi Arabia and elsewhere (18, 30–32). Moreover, diabetic patients in this study population were at higher risk of severe disease, consistent with previous studies (33, 34). According to Palaiodimos et al., risk of death is elevated among hospitalized diabetic patients with COVID-19 compared to non-diabetic individuals (35). The pathophysiology of this elevated risk could be attributed to a compromised immune system caused by chronic or transient hyperglycemia as well as chronic inflammation (36, 37).

Laboratory parameters can be important indicators and predictors of disease severity. In this study, high levels of urea, creatinine, potassium, LDH, D-dimer, and AST were all statistically significantly associated with severe cases of COVID-19. Several prior reports have indicated a link between low renal function and poor COVID-19 prognosis (38–41). In addition, D-dimer was recognized to be a key predictor of COVID-19 prognosis, with high D-dimer most likely being caused by acute lung damage or increased risk of thromboembolic consequences (42, 43). The effect of COVID-19 on the liver has been controversial, with some research linking elevated liver enzyme levels to infection severity, whereas others found no difference between mild and severe infections (44). Our results also highlighted serum LDH as an independent risk factor associated with severe cases of COVID-19. Similar results have been reported by previous studies (45–48); collectively, these findings emphasize the importance of monitoring serum LDH levels in COVID-19 patients upon admission.

The SARI chest X-ray severity scoring system was proposed by Taylor et al. in 2015 as a validated CXR scoring method that non-radiologists may use to assess patients with acute respiratory infections. Despite having lower sensitivity than a CT scan, this system can be used to radiologically assess a severe acute respiratory infection (14). Our study demonstrated that the early evaluation of patients with COVID-19 was statistically significantly aided by chest imaging. Notably, 75% of patients had a high SARI chest X-ray score (4-5), and this high score was statistically significantly associated with severe COVID-19 cases (*p* < 0.001). It has been reported previously that the radiologic severity index can be used to describe disease severity and aid in therapy planning (49). Thus, a modified grading method based on chest X-rays can be of benefit in determining COVID-19 severity, particularly in places with limited resources and expertise.

Antibiotics have been used to treat COVID-19 in up to 72% of cases worldwide (50–52). In the current study, the antibiotics most commonly used during hospital stays were azithromycin and cephalosporins, while the most frequently prescribed antiviral medication was favipiravir. Similar findings were reported previously among Bangladeshi patients with COVID-19 (52). Notably, one of the most interesting findings of the current study was the relationship between the administration of azithromycin and considerably reduced rates of mortality, ICU admission, and mechanical ventilation; in addition, azithromycin was more prescribed in severe COVID-19. The potential efficacy of azithromycin against SARS-CoV-2 infection is hypothesized to stem from multiple mechanisms of action, including increasing cellular pH to prevent virus entry, binding to and inhibiting the SARS-CoV-2 spike protein, reducing several inflammatory cytokines that have been shown to be major drivers of COVID-19 mortality, and finally treating secondary bacterial infections (53–55). Despite these facts, an Italian study with a large sample size (4,861) and propensity-matched controls of the same number, found that azithromycin was ineffective in treating SARS-CoV-2 infection, and raised concerns about the hazards associated with its inappropriate use (53).

In addition to azithromycin, this study found favipiravir and corticosteroids to be more utilized in patients with severe disease (*p* < 0.05). Moreover, these medications were reported with lower mortality, intubation, and ICU admission. Favipiravir has been identified as one of the most effective COVID-19 treatments when provided early in the disease’s course, since it can increase viral clearance and improve clinical outcome (56). Meanwhile, corticosteroids are recommended for patients with severe or critical COVID-19 (60). Notably, while steroids are helpful and lower mortality because of their beneficial regulatory effects against hyper-inflammation, they could also increase mortality by allowing more secondary infections (57–59). Moreover, since interleukin-6 inhibitors, vasopressors, carbapenems, and linezolid are frequently administered to critically ill COVID-19 patients who are already at a higher risk of poor outcomes, the observed associations between these drugs and poor outcomes may be due to their conditions rather than a direct effect of the treatments.

Our study’s strengths include data only from confirmed hospitalized COVID-19 patients in Saudi Arabia’s eastern province where there has been limited study among this community. Also, a three-step analysis ending in multivariate analysis was used which enhanced the methodological value and contribution. Furthermore, our study addressed the limitations of previously published papers by including the missing parameters such as laboratory parameters (8). It also looked at determinants of COVID-19 severity as well as other outcomes like ICU admission and mechanical ventilation requirement, rather than only death (9). As a result, this study sheds light on numerous parameters that may be utilized to construct a prediction model for evaluating the risk of severe COVID-19 and might assist healthcare practitioners manage patients, especially in locations with limited resources such as using the SARI chest X-ray scoring system which supported Satoto et al. finding (49). Despite these, there are some limitations that might affect the results of our study. First, data were collected from a single center over a short time and did not include pregnant women; as such, the generalizability of the results to the broader population is limited. Second, cross-validation was not conducted to overcome the overfitting problem; thus, future studies should consider using it to improve the robustness of model evaluations. Third, COVID-19 vaccinations had not yet been approved at the time of data collection, and new strains had not yet been detected, thus the association reported in this study may be altered. Moreover, no protective factors such as healthy diet, supplementation, or atopic conditions were included in a COVID-19 severity prediction model. Finally, we acknowledge the possibility that additional factors were not examined in this study which may also influence the severity of COVID-19. On this basis, further replication with independent cohorts is required.

## Conclusion

This study identified hypertension and diabetes mellitus as the most common comorbidities among COVID-19 patients in the eastern province of Saudi Arabia. The results highlighted a number of factors that can help predict the possibility of severe COVID-19, such as old age, diabetes mellitus, and high values for the laboratory parameters including urea, creatinine, potassium, LDH, D-dimer, and AST. After adjustment, only serum LDH was found to be an independent risk factor associated with severe COVID-19 infection. Finally, using azithromycin, corticosteroids, or favipiravir was statistically significantly associated with reduced mortality, ICU admission, and need for mechanical ventilation. These indicators can potentially be combined into a scoring system to identify high-risk individuals, identify priority groups for COVID-19 vaccination, facilitate better outcomes, and potentially develop preventative strategies.

## Data Availability

The original contributions presented in the study are included in the article/supplementary material, further inquiries can be directed to the corresponding author.
